# Transcriptional signatures of endothelial cells shape immune responses in cardiopulmonary health and disease

**DOI:** 10.1172/jci.insight.191059

**Published:** 2025-05-22

**Authors:** Elisabeth Fließer, Katharina Jandl, Shiau-Haln Chen, Mei-Tzu Wang, Jonas C. Schupp, Wolfgang M. Kuebler, Andrew H. Baker, Grazyna Kwapiszewska

**Affiliations:** 1 Otto Loewi Research Center, Lung Research Cluster, Medical University of Graz, Graz, Austria.; 2 Institute for Lung Health, Cardiopulmonary Institute, Member of German Lung Center, Justus-Liebig University, Giessen, Germany.; 3 Division of Pharmacology, Otto Loewi Research Center, Medical University of Graz, Graz, Austria.; 4 Centre for Cardiovascular Science, University of Edinburgh, Edinburgh, United Kingdom.; 5 Institute of Emergency and Critical Care Medicine, National Yang Ming Chiao Tung University, Taipei, Taiwan.; 6 Section of Pulmonary, Critical Care, and Sleep Medicine, Yale University School of Medicine, New Haven, Connecticut, USA.; 7 Department of Pulmonary and Infectious Diseases, Hannover Medical School, Hannover, Germany.; 8 Biomedical Research in End-Stage and Obstructive Lung Disease (BREATH), German Center for Lung Research BREATH, Hannover, Germany.; 9 Institute of Physiology, Charité-Universitätsmedizin, Berlin, Germany.; 10 German Center for Cardiovascular Research, Partner Site Berlin, Berlin, Germany.; 11 German Center for Lung Research, Associated Partner Site Berlin, Berlin, Germany.; 12 Department of Surgery and; 13 Department of Physiology, University of Toronto, Toronto, Ontario, Canada.; 14 Keenan Research Centre, St Michael’s Hospital, Toronto, Ontario, Canada.; 15 Department of Pathology, Cardiovascular Research Institute Maastricht, School for Cardiovascular Diseases, Maastricht University, Maastricht, Netherlands.

## Abstract

The cardiopulmonary vasculature and its associated endothelial cells (ECs) play an essential role in sustaining life by ensuring the delivery of oxygen and nutrients. Beyond these foundational functions, ECs serve as key regulators of immune responses. Recent advances in single-cell RNA sequencing have revealed that the cardiopulmonary vasculature is composed of diverse EC subpopulations, some of which exhibit specialized immunomodulatory properties. Evidence for immunomodulation includes distinct expression profiles associated with antigen presentation, cytokine secretion, immune cell recruitment, translocation, and clearance — functions critical for maintaining homeostasis in the heart and lungs. In cardiopulmonary diseases, ECs undergo substantial transcriptional reprogramming, leading to a shift from homeostasis to an activated state marked by heightened immunomodulatory activity. This transformation has highlighted the critical role for ECs in disease pathogenesis and their potential as future therapy targets. This Review emphasizes the diverse functions of ECs in the heart and lungs, particularly adaptive and maladaptive immunoregulatory roles in cardiopulmonary health and disease.

## Introduction

The cardiopulmonary vascular system is essential for sustaining life, as it not only facilitates gas exchange and nutrition, but also serves as a key platform for immune surveillance and response (1). Endothelial cells (ECs) play a pivotal role in this system. They secrete mediators implicated in thrombi formation and in maintenance of antithrombotic properties of the vasculature, present a crucial signaling compartment with other cell populations (e.g., immune cells, smooth muscle cells [SMCs]) (2–5) and regulate the vascular tone by secreting vasoactive mediators (endothelin-1, prostacyclin) (6).

Finally, ECs execute central immunomodulatory functions. By secreting chemokines and cytokines, expressing costimulatory adhesion molecules (VCAM-1, ICAM-1) and selectins (E selectin, P selectin), and acting as antigen-presenting cells, ECs can promote immune homeostasis via immediate and adaptive responses but also drive active and chronic inflammation (7). Importantly, advances in single-cell multi-omics profiling reveal that ECs of different tissues and organs exhibit a much greater immunomodulatory capacity beyond these traditional functions performed in their steady state (8–10). This Review aims to connect known functional properties of ECs in the lungs and heart with novel insights from single-cell RNA-Seq (scRNA-Seq) studies (as recapitulated in Table 1 and Table 2), with an emphasis on critical immunomodulatory roles and explores the therapeutic potential of targeting EC-driven maladaptive immune responses.

## Endothelial heterogeneity determines homeostatic cardiopulmonary system function

### ECs in the lung.

**Figure 1:**
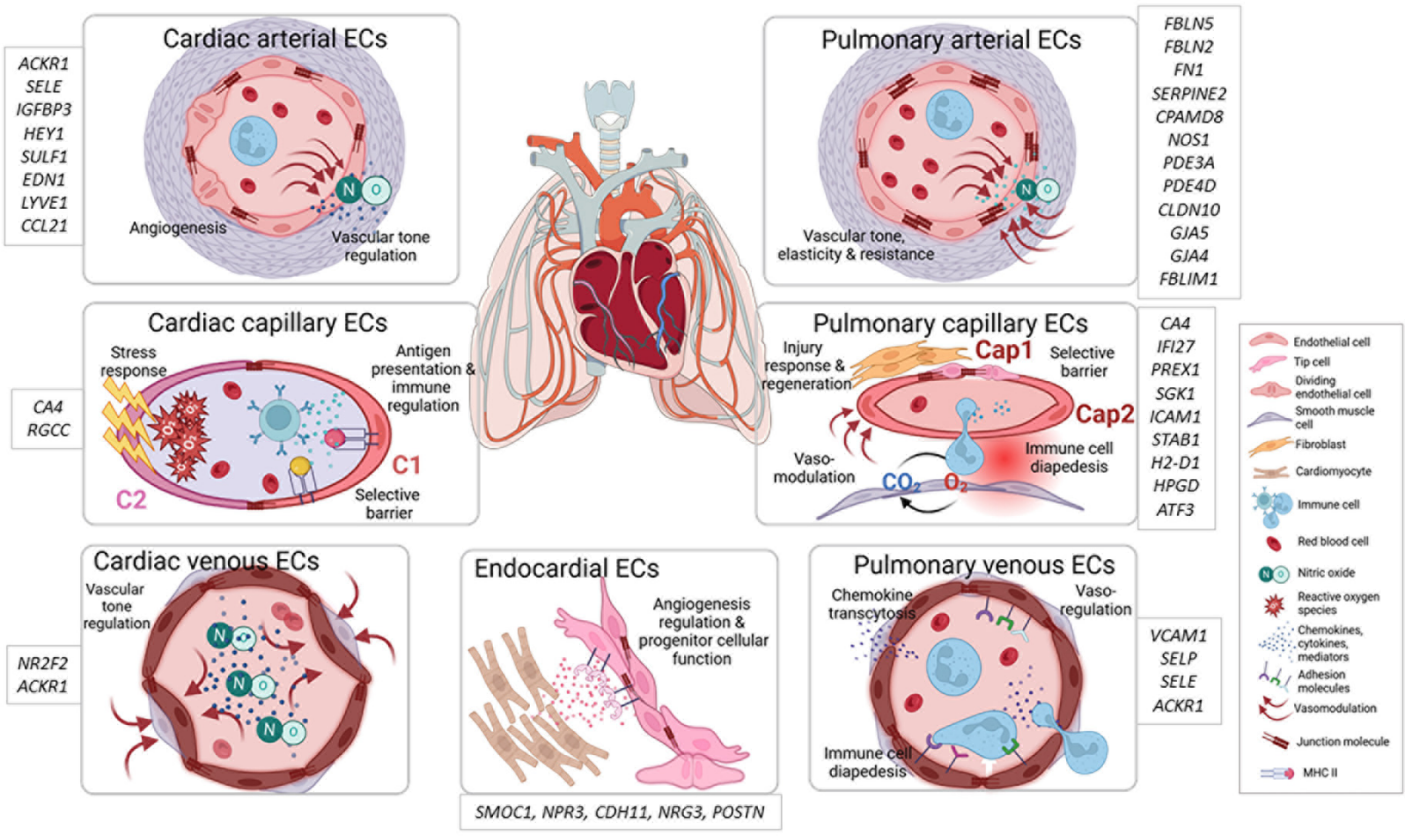
Cardiopulmonary EC heterogeneity in the steady state. Conceptual overview of cardiopulmonary EC heterogeneity in the steady state showing the main structure, functions, and signature genes of cardiac arterial, cardiac venous, cardiac capillary, endocardial, pulmonary arterial, pulmonary venous, and pulmonary capillary ECs. Cap1, general capillary EC; C1, capillary EC subcluster 1; C2, capillary EC subcluster 2; Cap2, aerocyte; NO, nitric oxide; O_2_, oxygen.

The lung is highly vascularized, with the capillary system making up a large part of the organ and closely interacting with epithelial, parenchymal, and immune cells (11, 12). The lung endothelium consists of vascular ECs, which are further classified as arterial, venous, and capillary ECs and of lymphatic ECs (9). Each EC subtype has specific immunological gene signatures within the pulmonary endothelium, which are linked to antigen presentation, immune cell recruitment, and cytokine secretion. This part of the Review underscores their importance in both protection from harmful threats but also in prevention of exaggerated inflammatory responses by maintaining immune tolerance (8, 9, 13, 14) (Figure 1). Of note, vascular ECs of the bronchial circulation are beyond the scope of this Review and are excellently reviewed elsewhere (15, 16).

Arterial ECs are pivotal in regulating vascular tone (17), which is reflected by a gene signature linked to strength/resistance and elasticity (*FBLN5*, *FBLN2*, *FN1)*, protease inhibitors (*SERPINE2*, *CPAMD8)*, and the NO pathway (*NOS1*, *PDE3A*, *PDE4D*) (9). Moreover, they are enriched in gap and tight junction genes (*CLDN10*, *GJA5*, *GJA4*, *FBLIM1*) (9).

Together with the basement membrane (BM) and alveolar epithelial cells, pulmonary capillary ECs build the main gas exchange unit (18). The nonfenestrated, continuous capillary endothelium possesses a highly restrictive barrier and is central in the regulation of immune cell migration and fluid fluxes (19, 20). Accordingly, capillary ECs express signature genes that are primarily associated with gas exchange (*CA4*), intracellular signaling, stress response, cell adhesion (*IFI27*, *PREX1*, *SGK1),* structural organization, and MHC class I (9).

Based on scRNA-Seq studies, capillary ECs are further divided into two subpopulations — the general capillary cells/gCap (Cap1) and the aerocytes/aCap (Cap2). The Cap1 transcriptome represents vasomotor regulation, antigen presentation, and progenitor function (8, 21). In mice, the unique progenitor potential of Cap1 is based on the regenerative activating transcription factor 3 (*Atf3*) (22). In contrast, ECs belonging to the Cap2 subpopulation exhibit an immunomodulatory signature (*ICAM1*, *STAB1*, *H2*-*D1*) and are the only EC population expressing 15-hydroxyprostaglandin dehydrogenase (*HPGD*), while lacking several common EC markers, such as *VWF* or *SELP* (8, 9, 21).

The venous endothelium functions as a vasoregulatory compartment, which is important for the ventilation-perfusion matching in the lung and facilitates immune cell infiltration (23). Concordantly, venous ECs exhibit transcriptomic signatures associated with motility and structural integrity and extracellular matrix (ECM) proteins as well as immune cell diapedesis and chemokine transcytosis (*VCAM1*, *SELP*, *SELE*, *ACKR1*) (9).

Lymphatic ECs, while essential for fluid balance and edema prevention, also play a critical role in adaptive immune responses through antigen and immune cell trafficking to lymph nodes (24). They are characterized by signature genes implicated in lymphangiogenesis, membrane integrity, and cell adhesion (9).

### ECs in the heart.

The heart is a highly vascularized organ that depends on ECs for structure, vasoregulation, and maintaining homeostasis through communication with immune cells and other cardiac cells (25). Cardiac ECs are divided into arterial, capillary, venous, endocardial, and lymphatic populations, each of which exhibit distinct functional properties shaped by location and transcriptional profile (Figure 1). All cardiac ECs are enriched in genes that regulate membrane transport and redox homeostasis, reflecting the highly oxidative microenvironment (26, 27).

Functionally, heart arterial ECs are central regulators of vascular contractility and thus coronary blood flow (28). Transcriptionally, they are divided into four subtypes (EC1–EC4) (10). EC1s are proinflammatory (*ACKR1*, *SELE*), while EC2s contribute to vascular development (*IGFBP3*, *HEY1*). EC3s are involved in ECM organization (*SULF1*, *EDN1*), and EC4s are responsible for neutrophil-mediated immune responses (*LYVE1*, *CCL21*) (10). Whether these populations are independent of each other or dynamically convertible needs additional investigation. Heart arterial ECs are also enriched in genes encoding vascular development and angiogenesis regulators (29–32).

Similarly, murine coronary artery ECs are also divided into 3 distinct clusters, with one implicated in immune responses, another in intercellular adhesion, and the third in growth and vasomodulation (33). This is accompanied by a spatial heterogeneity of the immune-modulatory clusters across coronary artery branches (33), indicating distinct local needs for immunoregulation in the steady-state heart.

Cardiac capillary ECs maintain a selective barrier, thereby facilitating gas exchange and immune cell trafficking (34). On the transcriptional level, they are marked by *CA4* and *RGCC* and can be divided into an antigen-presenting and a stress-responding EC population (35, 36). Correspondingly, a multiorgan study focusing on EC diversity in healthy adult mice revealed an IFN responsive cardiac capillary population, termed “IFN-activated ECs” (37).

Cardiac venous ECs are less well studied and characterized by expression of *NR2F2*, which regulates endothelial metabolism and development (38) and *ACKR1*, a regulator of innate immune responses (36, 39).

Endocardial ECs are central regulators of cardiomyocytes (40). Accordingly, their gene signature (*SMOC1*, *NPR3*, *CDH11*, *NRG3*, *POSTN)* (41–43) corresponds to vascular development, cellular structure, platelet activation, metabolism, and growth factor signaling. *POSTN* is further linked to regulation of vascular remodeling (44).

Only a few single-cell studies have explored molecular heterogeneity in adult cardiac lymphatics, yet these vessels play a key role in immune cell trafficking, preventing chronic inflammation and disease progression, and are characterized by *PROX1*, *TBX1*, and *PDPN* (45–47).

Taken together, the current state of knowledge on EC heterogeneity reinforces the cardiopulmonary ECs as a key component of immune regulation owing to their central location between different cell populations and ECM components as well as their complex signaling networks.

## Transition from endothelial homeostasis to activation

The cardiopulmonary endothelium maintains an antiinflammatory, antithrombotic, and barrier-protective phenotype, which is crucial for vascular homeostasis (48). Disruptions to homeostasis trigger transcriptional programs that drive metabolic shifts and alter EC functions, including junction stability, permeability, and vaso- as well as immunomodulation (49, 50). Beyond investigations of individual molecular pathways and molecules, emerging omics-based studies reveal a dynamic interconnection of the transcriptomic, metabolomic, and functional landscape of endothelial heterogeneity in health and disease. Details are summarized in Table 3 and Table 4 and will be explored in the next sections.

### Adaptations in the lung.

**Figure 2:**
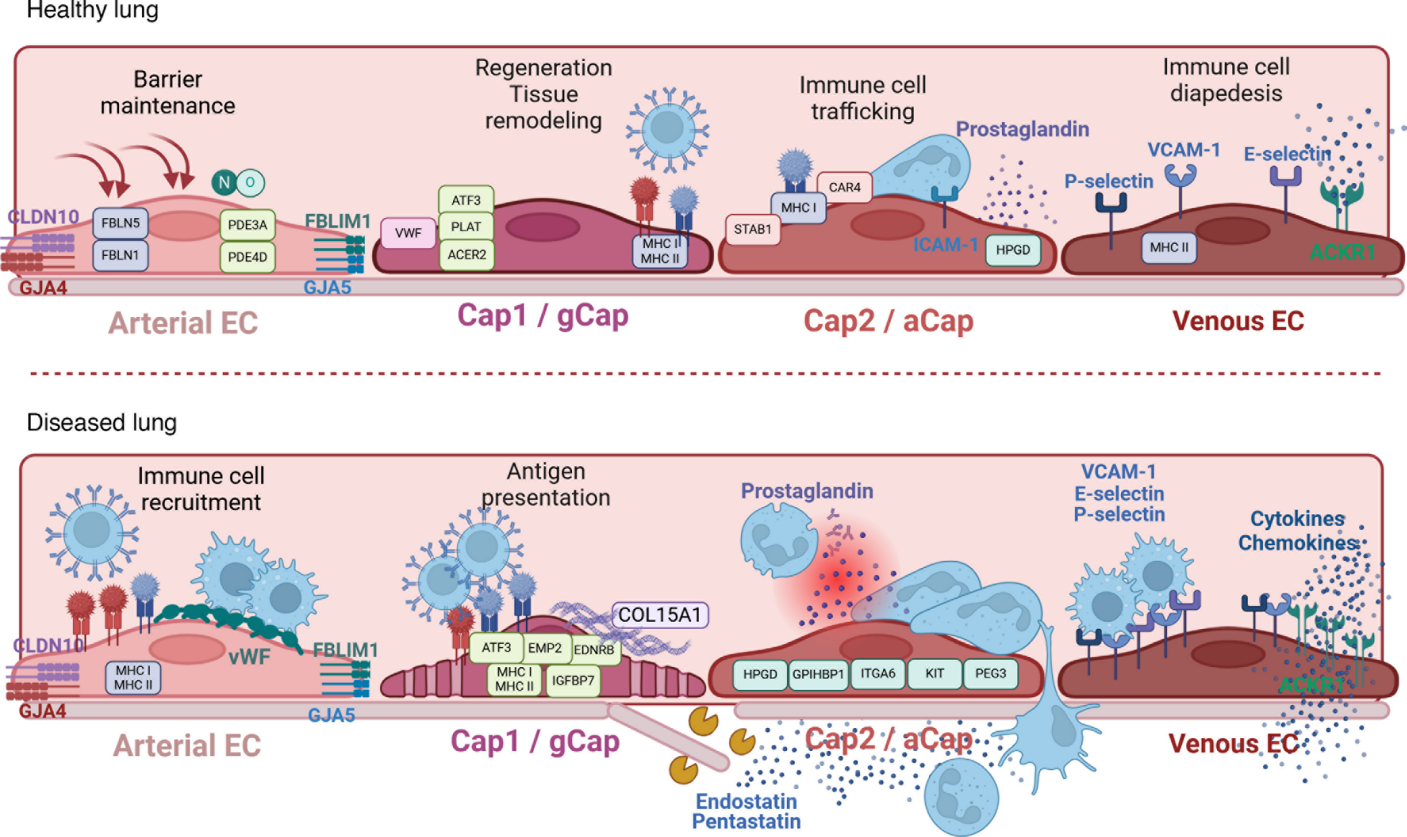
ECs as immune regulators in pulmonary health and disease. Conceptual overview of immunomodulatory functions in healthy and diseased lung ECs. The schematic represents signature genes and immune-regulatory molecules and mechanisms in the healthy pulmonary arterial, pulmonary venous, and pulmonary capillary ECs (top) and in their diseased state (bottom). aCap/Cap2, aerocyte; ACKR1, atypical chemokine receptor 1; CLDN10, Claudin 10; COL15A1, collagen type XV α 1; FBLIM1, filamin binding LIM protein 1; gCap/Cap1, general capillary cell; GJA4, gap junction protein α 4; GJA5, gap junction protein α 5; ICAM1, intercellular adhesion molecule 1; VCAM1, vascular cell adhesion molecule 1; vWF, von Willebrand Factor.

Lung endothelial injury is a key initiating event in vascular homeostasis disruption, setting off transcriptional and metabolic shifts that not only impair endothelial function, but also drive inflammatory immune responses. While the nature of this response varies by disease context, a shared feature is barrier function disturbance, leading to pathological remodeling and altered immune dynamics (Figure 2).

One of the most severe manifestations of EC dysfunction is acute respiratory distress syndrome (ARDS), where endothelial integrity loss results in increased vascular permeability, uncontrolled immune cell infiltration, and severe pulmonary edema (51). EC dysfunction in acute lung injury is closely linked to shifts in transcriptional phenotypes, particularly involving Cap1 and Cap2 EC populations (52). For example, injury due to exposure to hyperoxia in developing murine lungs increases proinflammatory Cap2, which impairs proper lung development (52). A similar pattern is observed in viral and chemically induced lung injuries. In murine models of influenza virus– or bleomycin-induced lung injury, Cap2 ECs were predominantly localized to areas of diffuse alveolar damage and exhibited upregulated antigen presentation and ECM remodeling genes (53). Additionally, the transcription factor *Atf3*, a Cap2 marker gene, was elevated in injured lungs, with its deficiency leading to EC loss and emphysema-like pathology in vivo (22, 54). Lung injury also triggers compensatory mechanisms that attempt to restore endothelial homeostasis. In LPS-treated mice, scRNA-Seq analysis identified two emerging EC subpopulations — one enriched in inflammatory response genes and the other linked to vascular regeneration (55). Interestingly, EC lineage tracing in different lung injury models revealed that venous ECs persistently proliferate and differentiate into capillary ECs (56). Whether these contribute to the transcriptionally altered, activated capillary pool after lung injury is currently being investigated.

A prominent example of this common endothelial injury-to-dysfunction progression is seen in SARS-CoV-2–mediated acute lung injury, where the endothelium exhibits a hyperactivated and hypercoagulatory phenotype, marked by elevated expression of adhesion molecules and inflammatory mediators (57). This hyperactivation is not limited to immune-endothelial crosstalk but also involves BM disruption. Patients with COVID-19 and non–COVID-19 ARDS exhibit high plasma levels of BM fragment endostatin, which contributes to endothelial barrier disruption in vitro, thereby potentially aggravating immune cell activation and migration (58). Targeting inflammatory hyperactivation has shown therapeutic promise. In SARS-CoV-2–infected hamsters, dexamethasone treatment stabilized EC integrity and ameliorated the course of the disease by reducing immune cell infiltration (59).

Heightened endothelial activation in lung injury is associated with metabolic shifts from the glycolysis/oxidative phosphorylation to alternative pathways (e.g., aerobic glycolysis) (60). In line, the glycolytic enzyme, 6-phosphofructo-2-kinase/fructose-2,6-biphosphatase 3 (PFKFB3), a key activator of aerobic glycolysis, was upregulated in lung ECs following LPS injury. Notably, EC-specific *Pfkfb3* deficiency has been shown to mitigate inflammation by reducing immune cell recruitment, highlighting the direct interplay between transcriptional metabolic adaptation, EC effector functions, and immunomodulation (61).

While acute EC dysfunction can be reversible in the best case, failure to resolve EC activation can lead to chronic fibrotic remodeling, such as found in progressive pulmonary fibrosis (PPF). PPF is characterized by parenchymal destruction and fibrogenesis that involves changes in the pulmonary endothelial and immune cell compartment (62, 63). In a rat model of bleomycin-induced pulmonary fibrosis, scRNA-Seq analysis identified EC subpopulation shifts, including expansion of a profibrotic cluster, which expressed genes related to cell migration, angiogenesis, ECM binding, and chemoattractants (64). These shifts were amplified by advanced age, causing the EC transcriptome to shift toward inflammation, fibrosis, and apoptosis, with a concomitant downregulation of common EC marker genes (65). Hyperactivated capillary and venous ECs persisted even after fibrosis resolution in an *Aplnr*-Cre-ERT lineage-tracing mouse model and continuously expressed gene signatures associated with immunoregulation, inflammation, and glycolysis (65).

Indeed, the bleomycin model is characterized by dynamic, time- and compartment-dependent shifts from an innate to an adaptive inflammatory profile within lung tissue and bronchoalveolar lavage fluid (66). The connection between ECs and immune cells in bleomycin-induced fibrosis was additionally supported by another study, where in vivo NKT cell activation partially restored the inflammatory profile in this model, leading to an increase in gene expression related to EC homeostasis, barrier function, and angiogenesis (67). Moreover, lymphatic ECs have been shown to facilitate clearance of immune cells and inflammatory mediators (46), and VEGF-3–mediated induction of lymphangiogenesis correlates with increased macrophage clearance and less fibrosis in the bleomycin model (68). In the complementary AP-1 transcription factor Fos-related antigen-2–overexpressing (Fra-2–overexpressing) model of systemic sclerosis-associated PPF (69, 70), pronounced immune infiltration (eosinophilia) in the lungs was associated with endothelial junction loss and worse disease outcome (71). Our recent study also highlighted the link between endothelial activation, inflammation, and tissue fibrosis (62). Detailed analysis of patients with PPF revealed an activated endothelium, characterized by pronounced vWF protein levels in the lung vasculature, which correlated with the presence of infiltrated immune cells and collagen deposition. This was accompanied by impaired barrier integrity of primary ECs in vitro (62). In fact, integrity loss of the gas-exchange unit, which consists of capillary ECs and juxtaposed AT1 cells, is a common early disease event in PPF (72). Moreover, scRNA-Seq data shows enrichment of *COL15A1*, *PLVAP*, and *VWA1* in capillary ECs of human fibrotic lungs, which is in line with histological replacement of PRX-positive capillary ECs by COL15A1-positive ECs. These are transcriptionally undistinguishable to fenestrated systemic-venous ECs (9, 73, 74). Within this histologically heterogeneous disease, COL15A1-positive ECs localize in dense fibrotic areas near fibrotic foci, suggesting that loss of capillary EC identity and integrity disrupts lung homeostasis and exacerbates fibrosis (74, 75).

An initial EC injury, alongside intrinsic predisposition, resulting in dysfunctional EC behavior, is also considered as a main trigger of the pathological remodeling cascade in pulmonary arterial hypertension (PAH). In the SU5416/hypoxia-induced PAH mouse model, arterial and capillary ECs were marked by a significant upregulation of genes involved in mitochondrial function, antigen presentation, angiogenesis, cell migration, vascular development, and shear stress response (76). Transcriptomic profiling of human PAH ECs further substantiated their metabolically active, immune-related state by revealing 629 differentially expressed genes related to mitochondrial function, ATP metabolism, oxidative phosphorylation, or respiration, further pointing toward a metabolically activated and immunomodulatory/inflamed endothelium (77).

In line, PAH is also associated with increased metabolic rates and elevated glycolysis, measured as increased levels of glycolytic and glutaminolysis-related enzymes, such as glucose transporter 1 (GLUT1), hexokinase 2 (HK2), lactate dehydrogenase A (LDHA), glutamate dehydrogenase 1 (GLUD1), and pyruvate dehydrogenase kinase (PDK) (14). These enzymes are linked to remodeling and proliferation in PAH (78). In addition, autophagy, another metabolic process, drives replacement of capillary ECs with hyperproliferating arterial ECs, thereby further worsening the disease (79). Besides, a sterol metabolism that is skewed toward increased oxysterol accumulation in ECs is associated with heightened endothelial immune-activation and worse disease outcome in both in vitro and in vivo PAH models (80). Moreover, KO or inhibition of endothelial malic enzyme 1 (ME1), which increases glucose uptake, protects against SU5416/hypoxia-induced PAH. This enzyme is upregulated in patients with PAH (81), demonstrating that EC metabolism is a promising therapeutic target.

Shear stress, which is present in PAH, is a potent regulator of endothelial metabolism (82) and can support the shift to an increased abundance of mesenchymal, inflammatory, and immune cell–like EC phenotypes (83). The detection of shear stress is associated with the mechanosensing capacity of ECs, which depends on finely tuned levels of the transcription factors Krüppel-like factor 2 (Klf2) and Klf4 levels (84, 85), both being disturbed/elevated in PAH (86). Moreover, disturbed flow can induce mechanosensing in ECs from the apical side, and increased subendothelial BM deposition activates mechanosensing programs from the basal side. Here, increased mechanoactivation of nuclear YAP translocation in ECs isolated from patients with PAH was linked to impaired barrier integrity (87). Indeed, the BM increasingly emerges as a hub for regulation of EC function. In that regard, endostatin (fragment from collagen XVIIIα1) is elevated in patients with PAH (88, 89), and pentastatin (fragment from collagen IVα5) induces EC injury and barrier dysfunction in PAH (90).

In chronic obstructive pulmonary disease (COPD), destruction of the alveolar cell–EC unit results in severe emphysema and lung function loss (91). A landmark study identified that inhibition of one of the main EC survival factors, VEGF, induced apoptosis and caused emphysema in rats (92). Furthermore, the loss of EC markers was associated with a more advanced clinical disease in patients (93). A deeper investigation of EC transcriptional changes revealed that in COPD, pulmonary capillary ECs upregulate gene signatures connected to cellular stress response and inflammatory signaling, while genes implicated in EC repair were downregulated (94).

### Adaptations in the heart.

**Figure 3:**
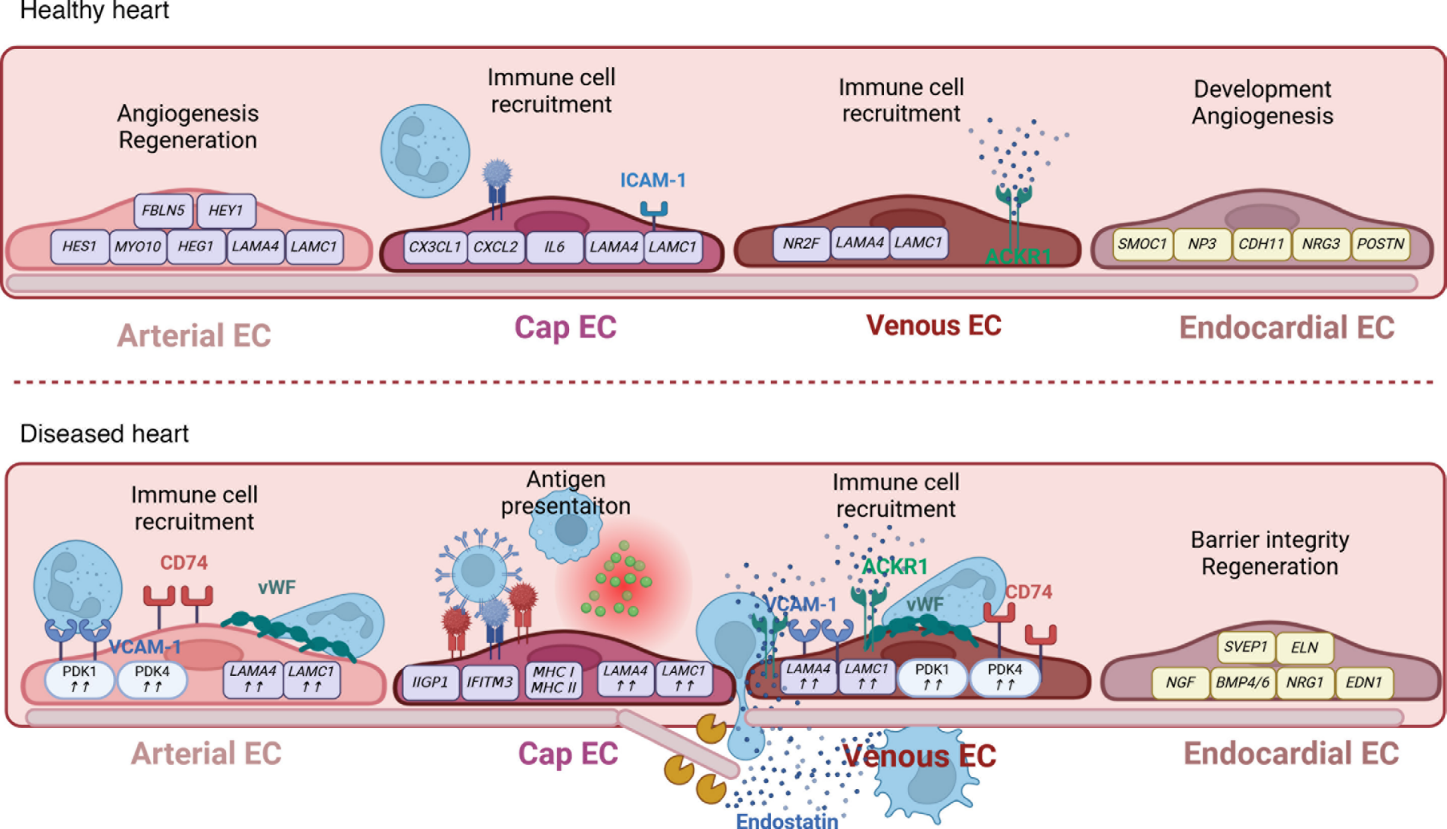
ECs as immune regulators in cardiac health and disease. Conceptual overview of immunomodulatory functions in healthy and diseased cardiac ECs. The schematic represents signature genes and immune-regulatory molecules and mechanisms in the healthy pulmonary arterial, pulmonary venous, pulmonary capillary, and endocardial ECs (top) and in their diseased state (bottom). ACKR1, atypical chemokine receptor 1; Cap EC, capillary EC; ICAM1, intercellular adhesion molecule 1; VCAM1, vascular cell adhesion molecule 1; vWF, von Willebrand Factor.

In the heart, ECs are central regulators of immunomodulation and immunological homeostasis (Figure 3), which is supported by the identification of immune cell–like EC subpopulations in the cardiac vasculature by scRNA-Seq (13, 83, 95, 96). Accordingly, ECs can perform macrophage-like functions, including cytokine secretion, phagocytosis, antigen presentation, and sensing of pathogen-associated molecular patterns and danger-associated molecular patterns (49, 50).

In atherosclerosis, a severe disease characterized by the accumulation of fibrous material in the arterial intima, metabolic EC reprogramming presents as upregulated glycolytic PDK1 and PDK4 expression and is linked to a proinflammatory, mesenchymal, and plaque-destabilizing gene signature (97, 98). Genes included in this signature are *VCAM1*, which has been implicated in monocyte infiltration, macrophage maturation, and foam cell transformation, and *VWF*, which facilitates inflammatory cell infiltration and exacerbation of atherosclerosis (99, 100). Moreover, MHC chaperone gene *CD74* is upregulated and has been linked to macrophage infiltration and endothelial-to-mesenchymal transition (EndoMT) in a murine lineage-tracing model of atherosclerosis (101). The disease is further driven by disturbed flow, which shifts the EC transcriptome from atheroprotective toward a proinflammatory, mesenchymal, and immune cell-like phenotype (83). This shift was confirmed in vitro, where disturbed flow induced endothelial integrin signaling and subsequent VCAM-1 and ICAM-1 expression, which enhances inflammatory cell infiltration and thus plaque formation (102, 103). Ultimately, the transcriptomic shift in atherosclerosis is further worsened by advanced age, which, by fueling the proinflammatory cytokine production and reduced NO, triggers plaque formation/expansion and tissue remodeling (104–106). Once more, these results confirm that closely interconnected metabolic and transcriptomic reprogramming provokes endothelial inflammation, immune cell infiltration, and overall worsening of the course of disease.

Human heart failure is another severe cardiac syndrome caused by myocardial injury, such as ischemic heart disease, myocardial infarction (MI), or cardiomyopathy, that leads to pronounced EC dysfunction and thus insufficient gas exchange (107). Here, scRNA-Seq analysis uncovered transcriptional shifts in endocardial and capillary ECs, leading to enrichment of pathways associated with nerve growth factor (NGF) signaling, IFN-γ signaling, antigen presentation, and disturbances in EC metabolism, phagosome function, and hedgehog signaling, which are important for vascular integrity maintenance (108–111). Moreover, scRNA-Seq analysis revealed the appearance of an immune-regulatory EC subpopulation in animal models of human heart failure and in MI (112). The gene expression profile of these ECs relates to cell proliferation, IFN responses, and immune regulation and is enriched in the regenerative phase of the injured murine heart. Myocardial injury is further enriched in signaling ligands with corresponding receptors on macrophages and T cells that mediate immune cell adhesion in vitro, as well as MHC genes, which are important for antigen presentation and thus a rapid immune response induction (112, 113). In vivo, stimulation of lymphangiogenesis in a post-MI animal model accelerated immune cell clearance, reduced inflammation, and ameliorated tissue remodeling and overall cardiac outcome (114, 115). Concordantly, promoting lymphangiogenesis via simultaneous delivery of lymphatic endothelial progenitor cells and VEGF-C effectively induced inflammatory cell clearance and repair of the infarcted myocardium in rats (68). A recent study also showed that transcription factor TBX-1 in cardiac lymphatic ECs facilitates post-MI repair by enhancing lymphangiogenesis and immunosuppression in mice (116). The feasibility of targeted induction of lymphangiogenesis in patients is still under investigation.

Moreover, as observed in various pulmonary disorders (see above), BM component and fragment signaling plays a crucial role in cardiac diseases. In ischemic cardiomyopathies, signaling by laminin, a major BM component important for EC integrity (117), is increased. This increase is largely driven by elevated expression of ligands LAMA4, LAMB1, and LAMC1 in disease. LAMA4 mutations are associated with heart failure, while LAMB1 expression is higher in a mouse MI model (117, 118) and enhances fibrosis in heart pathologies via EndoMT (119). Additionally, BM degradation during cardiac injury releases bioactive matrikine fragments, which are implicated in EC barrier disruption, cytokine secretion, and immune cell activation, as evaluated in vitro and in vivo (120). Thus, the presence of skewed levels of BM components/fragments may significantly exacerbate the course of cardiomyopathies.

These global transcriptomic and metabolic changes in cardiopulmonary ECs point toward a pathological endothelium, which might provoke functional and physiological abnormalities.

Metabolomic and transcriptomic studies are an important tool to interpret cell functions and establish a link to underlying molecular signaling pathways and biological processes. Metabolic shifts underlie a complex interplay of extrinsic mechanisms, like altering the supply of EC metabolites and nutrients, and intrinsic mechanisms, such as altering the expression of genes involved in glycolysis or oxidative phosphorylation. Additionally, transcriptomic analyses offer insights into gene expression but do not always correlate with cellular functions due to posttranscriptional modifications, protein synthesis, and cellular interactions. Methodological limitations, such as bulk RNA-Seq averaging expression across a sample, must be considered. scRNA-Seq, while powerful for addressing this limitation and expanding our capacity in understanding cellular heterogeneity, also suffers from limitations such as cell composition biases. Differential cell loss during dissociation, preferential capture of certain cell types, and limitations in sequencing depth can all lead to inaccurate representation of rare cell populations and skewed interpretations of tissue heterogeneity. For example, EC isolation for scRNA-Seq using flow cytometry may result in underrepresentation of aerocytes/Cap2, as they lack certain common EC markers. In general, the transcriptomic and metabolic adaptations need to be linked to molecular pathways implicated in the inflammatory and regenerative cascade in order to understand their contribution to disease pathogenesis and to uncover novel therapeutic approaches (see below).

## The role of EndoMT in cardiopulmonary health and disease

EndoMT refers to the loss of common EC markers with a concomitant gain of mesenchymal markers and, thus, transformation of ECs from a barrier-forming, antiinflammatory phenotype into a mesenchymal-like, contractile, and migratory phenotype (121, 122). Although EndoMT is a crucial process in heart development (123) and increasingly evident in several cardiopulmonary diseases, it is still poorly defined with regard to the combination of markers, the expression strength (partial/complete loss of EC markers), and the transient or irreversible nature. Longitudinal studies using lineage tracing and in-depth transcriptomics revealed inflammation as a crucial driver of EndoMT and currently advance the state of knowledge on EndoMT in the cardiopulmonary system (124). Lineage-tracing studies using Cdh5-CreERT2-TdTomato mice in a model of SU5416/hypoxia-induced PH revealed that only a small proportion (<1%) of tomato-positive ECs coexpress mesenchymal markers under both normoxic and hypoxic conditions (76). Other studies have reported slightly higher incidences of EndoMT in PAH. vWF-α-SMA double-positive cells have been detected in up to 5% of pulmonary vessels in the SU5416/hypoxia mouse model (125), and tracing EndoMT in Cdh5-Cre/GFP mice in response to SU5416/hypoxia revealed slightly higher percentages of double-positive cells (126). Moreover, lineage tracing of ECs in an Aplnr-Cre-ERT-EGFP mouse model of bleomycin-induced lung fibrosis revealed only a transient coexpression of the reporter fluorophore with mesenchymal marker genes *Col1a1*, *Acta2*, and *Fn1* at the peak of fibrosis (65). In the tobacco smoke–induced murine emphysema and COPD model, the vasculature exhibited increased expression levels of myofibroblast marker S100A4 (127). Although, elevated S100A4 may indicate EndoMT presence in COPD lungs, a thorough coexpression analysis with EC markers and lineage tracing studies is required.

In the heart, scRNA-Seq and bulk RNA-Seq analysis of ECs following MI in the lineage-tracing Cdh5-CreERT2 mouse model revealed that ECs acquire a transient mesenchymal phenotype 3 to 7 days after injury that disappeared after 14 days. The gene signature of the mesenchymal EC cluster was linked to ECM organization, PDGF binding, collagen synthesis, and organization, implying a role in EC migration and revascularization during tissue regeneration (128). Another study also showed a stage-specific role of ECs in ischemic cardiac injury. At day 2 after MI, cardiac ECs adopt a proinflammatory and proproliferative phenotype, which is accompanied by suppression of EC homeostatic genes (129). During the scar formation phase, ECs shift toward a proangiogenic, mesenchymal phenotype. Notably, permeability regulator *Plvap* and adhesion molecules *Sele* are upregulated both early after MI and during scar formation, reflecting persistent EC activation, potentially driving chronic inflammation (129). Moreover, in vitro histone deacetylase 9 (HDAC9) inhibition sustained EC marker expression and prevented mesenchymal transition, and HDAC9 KO in atherosclerosis-prone mice reduced plaque area, linking EndoMT to atherosclerosis progression (130).

Although EndoMT has been investigated in different animal models, its definite presence as well as contribution to disease remains unclear. scRNA-Seq results are frequently challenged by the process of distinguishing between stable cell clusters and transient states, as well as spurious and biologically meaningful clusters, all of which may affect our understanding of EC states and functions. Moreover, the loss of spatial information in scRNA-Seq can lead to an incomplete understanding of EC characteristics and interactions with other cells, such as SMCs within specialized pathogenic niches. If identity loss, and subsequent EC functional decline, such as barrier disruption, occurs and persists, it could be another EC-derived trigger for chronic inflammation. However, whether EndoMT represents a permanent phenotypic switch or a transient response to environmental conditions needs further investigation.

## The cardiopulmonary endothelium as an integrated unit

The heart and lung vasculature and their associated ECs share an intricate relationship by functioning as a cardiopulmonary unit (131–133). Cardiovascular diseases and chronic lung conditions are often treated as distinct illnesses, despite the intimate anatomical, mechanical, hemodynamic, and neurohumoral association. The consequences of maladaptive endothelial changes in one organ can propagate immune/inflammatory responses in the other, underscoring their functional integration as a singular immunological system.

For example, in left heart failure the inability of the ventricle to pump a physiological stroke volume causes a progressive pressure build-up in the pulmonary circulation, which drives the development of cardiogenic lung edema, and chronically leads to the development of pulmonary hypertension with left heart disease (134, 135). Elegant electron microscopic studies have shown that, in rabbit lungs, the earliest physical disruption of the capillary endothelium occurs at capillary transmural pressures of 24 mmHg. Notably, the stress-inducing threshold varies as a function of organism size and/or age, with high threshold pressures in equine lungs and conversely low thresholds in newborn rabbits (136, 137); however, lower pressure elevations suffice to increase lung capillary permeability (138, 139). This pressure-induced permeability increase has been linked to the mechanosensitive cation channel TRPV4, which accordingly depicts an important therapeutic target to treat acute cardiogenic edema (140, 141). Chronic pressure stress due to congestive left heart failure causes extensive remodeling of the alveolar-capillary membrane, as observed in pacing-induced heart failure in dogs or in an aortic banding model in guinea pigs, leading to the loss of TRPV4 in lung ECs (142–144). These adaptations decrease capillary permeability and thus protect the lung from excessive cardiogenic edema in chronic heart failure (143, 145).

In line with alveolocapillary membrane remodeling, heart failure is typically associated with a reduced pulmonary diffusion capacity for carbon monoxide and systemic hypoxemia (146). It is presently, however, unclear whether impaired pulmonary oxygenation is due to an increased alveolocapillary diffusion barrier, impaired ventilation-perfusion matching, or lung alveolar capillarization loss (146). Importantly, the alveolar-capillary maladaptation is paralleled by extensive pulmonary arterial and venous remodeling and stiffening (147, 148) as well as SMC osteogenic or chondroid transdifferentiation (149, 150).

Some evidence suggests that endothelial maladaptation is more pronounced in heart failure with reduced ejection fraction (HFrEF; left ventricular ejection fraction [LVEF] <40%) as compared with heart failure with preserved ejection fraction (HFpEF; LVEF ≥50%) (151, 152). However, EC dysfunction was also shown in HFpEF, and direct disease comparisons are cautioned, owing to differences in disease duration and severity. Notably, the acute and chronic responses to left heart failure are largely orchestrated by the pulmonary endothelium. In response to changes in hemodynamic forces, ECs not only cause acute vascular permeability, but also trigger responses in juxtaposed cells, such as an impairment of the alveolar-epithelial fluid reabsorption in acute cardiogenic edema (153). Additionally, ECs promote vascular remodeling in chronic left heart disease by extensive ECM deposition, which drives SMC proliferation (154).

Emerging evidence also highlights a central role for immune activation and inflammation in these adaptations (155, 156). Chronic low-grade inflammation may exacerbate EC dysfunction by activating immune cells and promoting proinflammatory cytokine release (156, 157). In animal models, inflammatory responses translocating from the lungs to the systemic circulation have not only been confirmed as predictors of cardiovascular diseases, but have also been implicated in their pathogenesis (158). Moreover, the innate immune system contributes to pulmonary and cardiac remodeling through macrophage infiltration and activation of inflammatory pathways, which further promote tissue remodeling (155, 157). The inflammatory environment and endothelial maladaptation may create a loop where hypoxemia triggers immune activation and metabolic stress, worsening cardiopulmonary decline. The cardiopulmonary endothelium plays a key role in integrating vascular, immune, and metabolic signals, presenting potential therapeutic targets to disrupt this feedback loop.

Right heart failure, which has been less studied, occurs due to a mismatch between the right ventricle’s ability to pump blood and increased pressure from PAH, although pressure-independent PAH components likely also contribute to the disease development (159). For instance, pulmonary vascular EC dysfunction in PAH fuels immune-mediated vascular remodeling and drives aberrant immune responses, such as cytokine storm, hyperactivation, and hypercoagulation (160–162). Impairment of the endothelial barrier also allows an influx of blood components, which further stimulate a proinflammatory environment (163–165). The precise mechanisms by which pulmonary inflammation affects the right ventricle, independent of hemodynamic stress, remain incompletely understood. Thus, the distinction between hemodynamic strain and systemic inflammatory crosstalk lies at the heart of heart-lung interactions in PAH and warrants further investigation.

## Current applications and prospective therapies implicated in EC-mediated immune responses

Although EC-targeted treatment options for cardiopulmonary diseases are scarce, the new paradigm of immunomodulatory ECs as determinants of health and disease makes immune-vascular crosstalk interesting for potential therapeutic intervention. Indeed, current PF medications influence EC and immune cell behavior. Pirfenidone and nintedanib, the only two approved antifibrotic drugs, work to some extent via the endothelial VEGF/VEGFR-2 axis (166, 167) and prevent EC junction disassembly (168). Moreover, the phase III PF therapeutic nerandomilast (BI 1015550), a phosphodiesterase PDE4B inhibitor, suppresses the expression of endothelial adhesion molecules (169) and the administration of immunosuppressing compound tacrolimus attenuated both vascular and parenchymal remodeling in the rat model (170). These studies indicate that interfering with the EC–immune cell interaction cascade in lung fibrosis might be a crucial therapeutic avenue for the deadly disease. Furthermore, strong antiinflammatory corticosteroids commonly used in COPD inhibit proinflammatory cytokines and mediator production (prostaglandins, leukotrienes) in ECs and reduce adhesion molecule expression. The steroids also prevented barrier breakdown and edema formation in perfused rat lungs (171–175).

Patients with PAH are commonly treated with vasodilatory prostacyclin analogs that restore homeostasis and function by inhibiting the expression of EC surface selectins and adhesion molecules (176–179). Similarly, bosentan downregulates ICAM-1 on ECs (179), and sotatercept regulates EC proliferation and vascular remodeling in the SU5416/hypoxia rat model (180, 181). Immunomodulatory intervention studies using TNF-α pathway inhibitors or immune cell–targeting antibodies have also shown therapeutic potential (182–188). Other ongoing phase II or phase III trials of treatments that interfere with the EC–immune cell interaction (e.g., tacrolimus, imatinib, empagliflozin) cascade are currently ongoing (181).

In heart failure therapy, angiotensin-converting enzyme inhibitors can reduce oxidative stress and increase bradykinin levels to improve EC function (189). Statins have demonstrated good results in reducing inflammation (190, 191). Recently, a phase III autologous CardiAMP trial delivered autologous bone marrow cells to the heart of patients with ischemia-associated and medically refractory HFrEF (192), which stabilized EC progenitor function and regulated angiogenesis, oxidative stress, inflammation, and apoptosis (193). Some preclinical trials disclosed the potential role of immune checkpoint signaling in pathological processes of heart failure (194), where inhibition or deficiency of PDL-1 enhanced cardiomyopathy and allograft rejection in mice (195). Furthermore, inhibition of T cell–specific ICOS or EC-specific ICOS ligand prevented cardiac fibrosis development in a preclinical heart failure and myocarditis models (196, 197). Therefore, approved therapeutics and currently ongoing studies underpin the vast potential of targeting maladapted EC-mediated immune responses and EC–immune cell niches to ameliorate the progression and improve the outcome of various cardiopulmonary diseases.

## Conclusions

Advances in scRNA-Seq and imaging technologies have revealed substantial heterogeneity among cardiopulmonary ECs within different vascular compartments of both the heart and lungs. The identification of EC subpopulations with immunomodulatory phenotypes and heightened immunoregulatory functions suggests these specialized cells act as gatekeepers, creating so-called “hot spots” for immune cell interaction and translocation. This dynamic EC–immune cell interaction is essential for vascular homeostasis but also contributes to acute and chronic diseases. Whether and to what extent transcriptomic reprogramming occurs in lung and heart diseases and how EC subpopulations may shift from maintaining tissue homeostasis to actual drivers of inflammation, tissue damage, and severe pulmonary pathologies is still under investigation.

Although the value of RNA-Seq data is undeniable, the true significance of this approach emerges only when it is combined with physiological principles. Thus, integrating different transcriptomic technologies and epigenomics, proteomics, and metabolomics as well as cutting-edge in vitro and in vivo models will improve our understanding of maladaptive immunoregulatory shifts in the endothelium. Deciphering these shifts and understanding underlying molecular mechanisms and cellular niches involved in disturbed inflammatory crosstalk will reveal therapeutic targets for treating cardiopulmonary diseases, such as PAH, heart failure, and lung disorders.

## Figures and Tables

**Table 1:**
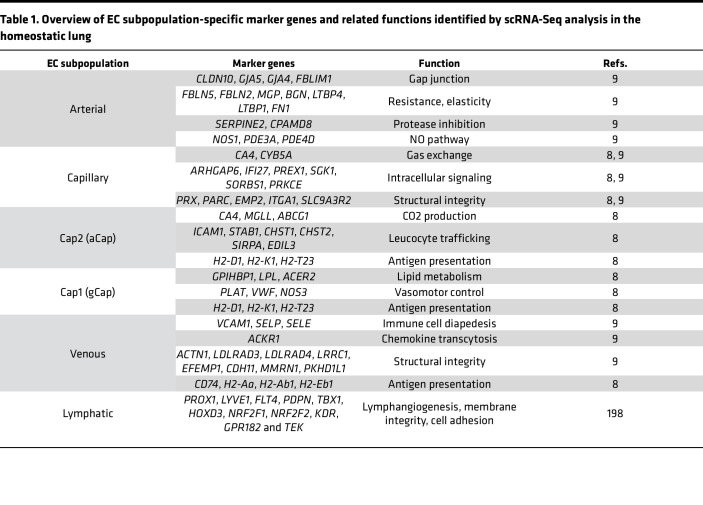
Overview of EC subpopulation-specific marker genes and related functions identified by scRNA-Seq analysis in the homeostatic lung

**Table 2:**
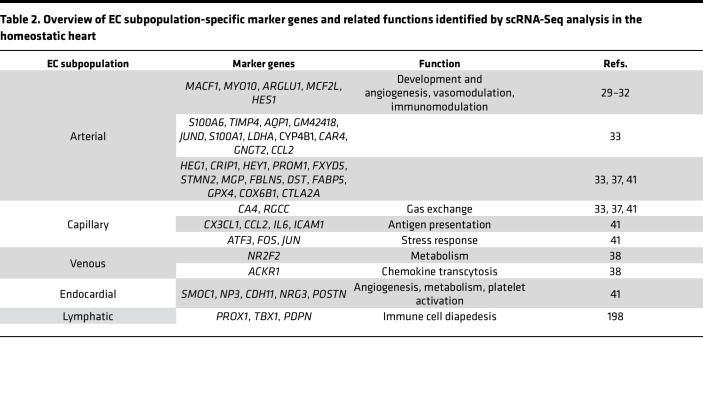
Overview of EC subpopulation-specific marker genes and related functions identified by scRNA-Seq analysis in the homeostatic heart

**Table 3:**
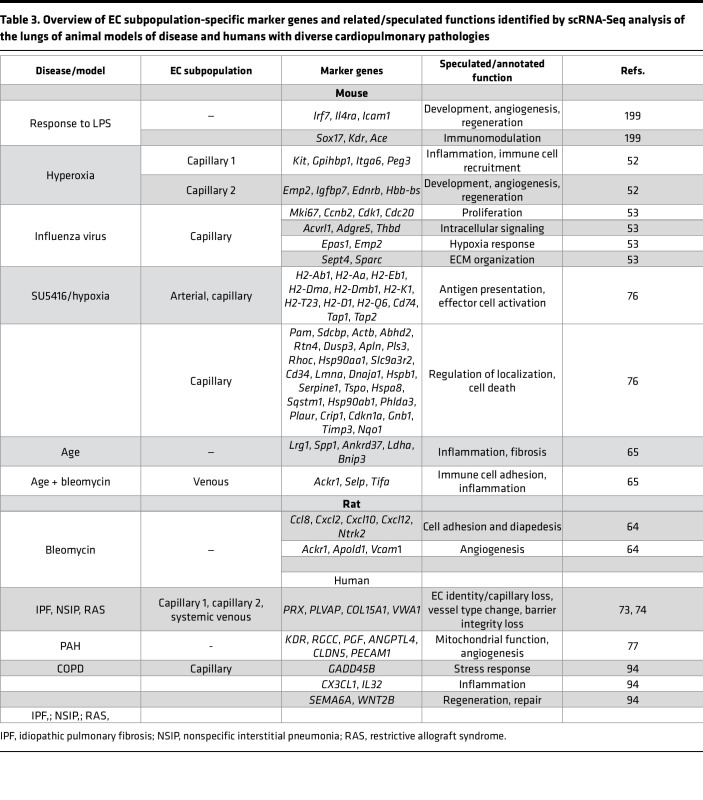
Overview of EC subpopulation-specific marker genes and related/speculated functions identified by scRNA-Seq analysis of the lungs of animal models of disease and humans with diverse cardiopulmonary pathologies

**Table 4:**
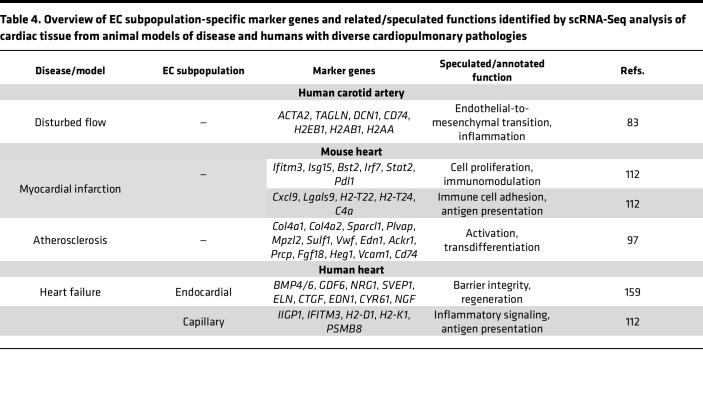
Overview of EC subpopulation-specific marker genes and related/speculated functions identified by scRNA-Seq analysis of cardiac tissue from animal models of disease and humans with diverse cardiopulmonary pathologies

## Version 1 05/22/2025

Electronic publication
